# Anti-Tumor Effect against Human Cancer Xenografts by a Fully Human Monoclonal Antibody to a Variant 8-Epitope of CD44R1 Expressed on Cancer Stem Cells

**DOI:** 10.1371/journal.pone.0029728

**Published:** 2012-01-17

**Authors:** Kazue Masuko, Shogo Okazaki, Mayumi Satoh, Goh Tanaka, Tatsuya Ikeda, Ryota Torii, Eri Ueda, Takashi Nakano, Masaaki Danbayashi, Tomoyo Tsuruoka, Yoshiya Ohno, Hideki Yagi, Noritsugu Yabe, Hideaki Yoshida, Tomoyuki Tahara, Shiro Kataoka, Taichi Oshino, Takayuki Shindo, Shin-ichiro Niwa, Takatsugu Ishimoto, Hideo Baba, Yoshiyuki Hashimoto, Hideyuki Saya, Takashi Masuko

**Affiliations:** 1 Cell Biology Laboratory, Department of Pharmaceutical Sciences, School of Pharmacy, Kinki University, Higashiosaka-shi, Osaka, Japan; 2 Kohjin Bio Co., Ltd., Saitama, Japan; 3 Kyowa Hakko Kirin Co., Ltd., Chiyoda-ku, Tokyo, Japan; 4 Link Genomics, Inc., Chuo-ku, Tokyo, Japan; 5 Department of Gastroenterological Surgery, Graduate School of Medical Science, Kumamoto University, Honjo, Kumamoto, Japan; 6 Tohoku University, Sendai, Japan; 7 Division of Gene Regulation, Institute for Advanced Medical Research, School of Medicine, Keio University, Shinjuku-ku, Tokyo, Japan; 8 Pharmaceutical Research and Technology Institute, Kinki University, Higashiosaka-shi, Osaka, Japan; Duke-National University of Singapore Graduate Medical School, Singapore

## Abstract

**Background:**

CD44 is a major cellular receptor for hyaluronic acids. The stem structure of CD44 encoded by ten normal exons can be enlarged by ten variant exons (v1-v10) by alternative splicing. We have succeeded in preparing MV5 fully human IgM and its class-switched GV5 IgG monoclonal antibody (mAb) recognizing the extracellular domain of a CD44R1 isoform that contains the inserted region coded by variant (v8, v9 and v10) exons and is expressed on the surface of various human epithelial cancer cells.

**Methods and Principal Findings:**

We demonstrated the growth inhibition of human cancer xenografts by a GV5 IgG mAb reshaped from an MV5 IgM. The epitope recognized by MV5 and GV5 was identified to a v8-coding region by the analysis of mAb binding to various recombinant CD44 proteins by enzyme-linked immunosorbent assay. GV5 showed preferential reactivity against various malignant human cells versus normal human cells assessed by flow cytometry and immunohistological analysis. When ME180 human uterine cervix carcinoma cells were subcutaneously inoculated to athymic mice with GV5, significant inhibition of tumor formation was observed. Furthermore, intraperitoneal injections of GV5markedly inhibited the growth of visible established tumors from HSC-3 human larynx carcinoma cells that had been subcutaneously transplanted one week before the first treatment with GV5. From *in vitro* experiments, antibody-dependent cellular cytotoxicity and internalization of CD44R1 seemed to be possible mechanisms for *in vivo* anti-tumor activity by GV5.

**Conclusions:**

CD44R1 is an excellent molecular target for mAb therapy of cancer, possibly superior to molecules targeted by existing therapeutic mAb, such as Trastuzumab and Cetuximab recognizing human epidermal growth factor receptor family.

## Introduction

CD44 is a type I cell-surface glycoprotein, which functions as the major cellular adhesion molecule for hyaluronic acids [1]–[3]. Standard CD44 (CD44s) encoded by the ten normal exons (ex1-5 and ex16-20) can be enlarged by the inserts encoded by various combinations of variant exons (ex6-15 or v1-v10) of CD44 by alternative splicing [3], [4]. Although the physiological significance of the alternative splicing of CD44 remains unclear, some variant CD44 (CD44v) molecules were reported to be over-expressed in various malignancies of rodent and human systems [5]–[8].

Among many CD44v, CD44R1 [7], [8] having an inserted region encoded by v8 (ex13), v9 (ex14) and v10 (ex15) exons is selectively expressed in various human epithelial cancers. For example, CD44R1 mRNA is elevated in human colon, bladder, lung, larynx and breast cancers [8], and immunohistological analysis (IHA) also revealed that CD44R1 protein was over-expressed in lung pleural samples compared with that in adjacent normal tissues, using rabbit polyclonal antibodies raised against recombinant CD44 protein [8]. Furthermore, we have recently demonstrated that mouse homolog of human CD44R1 is expressed in precancerous regions, possibly containing cancer stem cells (CSCs) or tumor-initiating cells, during mouse gastric carcinogenesis [9], [10].

However, since specific fully human monoclonal antibodies (mAb) recognizing the extracellular domain of human CD44R1 expressed on living tumor cells have not been available until now, precise evaluation of the therapeutic effect of anti-CD44R1 mAb on human malignancies remains to be undertaken. In this study, we report the growth inhibition of human cancer xenografts in athymic mice by locally or systemically administered fully human mAb recognizing CD44R1, and discuss the specificity, anti-tumor mechanisms and usefulness of fully human anti-CD44R1 mAb in cancer therapy.

## Results and Discussion

CD44, which binds hyaluronates, is a credible marker molecule for CSCs [11]–[16], and is significantly involved in the metastasis of tumor cells [16]–[19]. Thus, CD44 is considered to be a promising molecular target for cancer therapy using mAb. Since CD44s is expressed in various normal tissues [20], we have focused on tumor-selective splice-variant CD44v proteins. Among over 1000 theoretically possible splice-variant CD44v proteins [21], CD44R1 having the insert coded by v8, v9 and v10 exons is selectively expressed on various epithelial cancer cells [8].

We have recently prepared five anti-human CD44 fully human IgM mAb (MV1 against CD44s, and MV2, MV3, MV4 and MV5 against CD44R1) from cell fusions between mouse myeloma cells and spleen cells of Kirin-Medarex (KM) mice [22] immunized against recombinant human CD44 proteins produced in *Escherichia coli*. In this paper, we present the preparation of class-switched anti-CD44R1 human IgG mAb (GV5), specificity of MV1, MV5 and GV5, and *in vivo* and *in vitro* anti-tumor effect of GV5.

### Fully human IgM and IgG mAb against human CD44 proteins were produced

**Figure 1:**
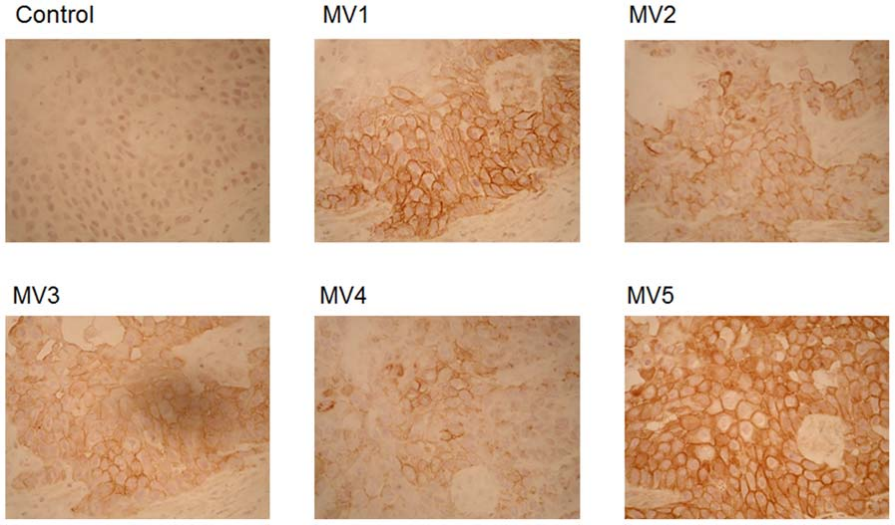
Immunoperoxidase staining of human xenografts developed in athymic mice with anti-CD44 fully human IgM mAb. Tissue sections of human ME180 tumors developed in athymic mice were fixed with PFA, and were sequentially incubated with human mAb, species-specific biotinylated anti-human IgG + IgM (H+L), ABC reagent and substrate solution containing DAB and H_2_O_2_. Nuclei were stained with hematoxylin.

**Figure 2:**
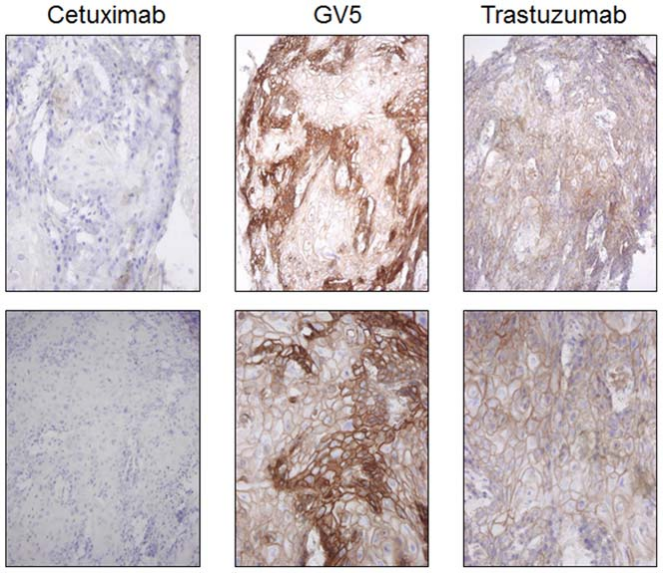
Immunoperoxidase staining of human xenografts developed in athymic mice with an anti-CD44R1 fully human IgG mAb (GV5). Tissue sections of human ME180 tumors developed in athymic mice were fixed with cold acetone, and were sequentially incubated with primary mAb, species-specific biotinylated anti-human IgG Fcγ, ABC reagent and substrate solution containing DAB and H_2_O_2_. Nuclei were stained with hematoxylin. Upper or lower panels respectively show low or high magnification of the stained tissues.

Five anti-human CD44 fully human IgM mAb (MV1, MV2, MV3, MV4 and MV5) were produced against a recombinant CD44 (R1a; Δex5-v8-v9-v10-Δex16) protein produced in *Escherichia coli* [8]. MV1 reacted with RH7777 rat hepatoma cells expressing CD44s or CD44R1, and MV2, MV3, MV4 and MV5 reacted specifically with RH7777 cells expressing CD44R1 (data not shown). To evaluate the reactivity of human mAb with *in vivo* tumors, we performed IHA (**Fig. 1**). MV1 and MV5 definitely stained cell membranes of *in vivo* tumor from ME180 human uterine cervix cancer developed in athymic mice, and CD44R1 was heterogeneously expressed in human cancer xenografts, although MV2, MV3 and MV4 showed relatively weak reactions compared with MV5 (**Fig. 1**). Therefore, we have reshaped (class-switched) MV5 human IgM to GV5 human IgG. Reactivity of GV5 with *in vivo* tumors from ME180 was also positive at the cell membrane of these tumor cells (**Fig. 2**), and CD44R1 was heterogeneously expressed in the tumor as in the case with the staining of the ME180 tumor by MV5. In contrast, the expression of HER2 recognized with Trastuzumab was relatively uniform in the ME180 tumor, and the expression of CD20 recognized by Rituximab was completely negative.

### Specificity and epitope of fully human MV1 and MV5 IgM, and class-switched GV5 IgG mAb against CD44 were determined

**Figure 3:**
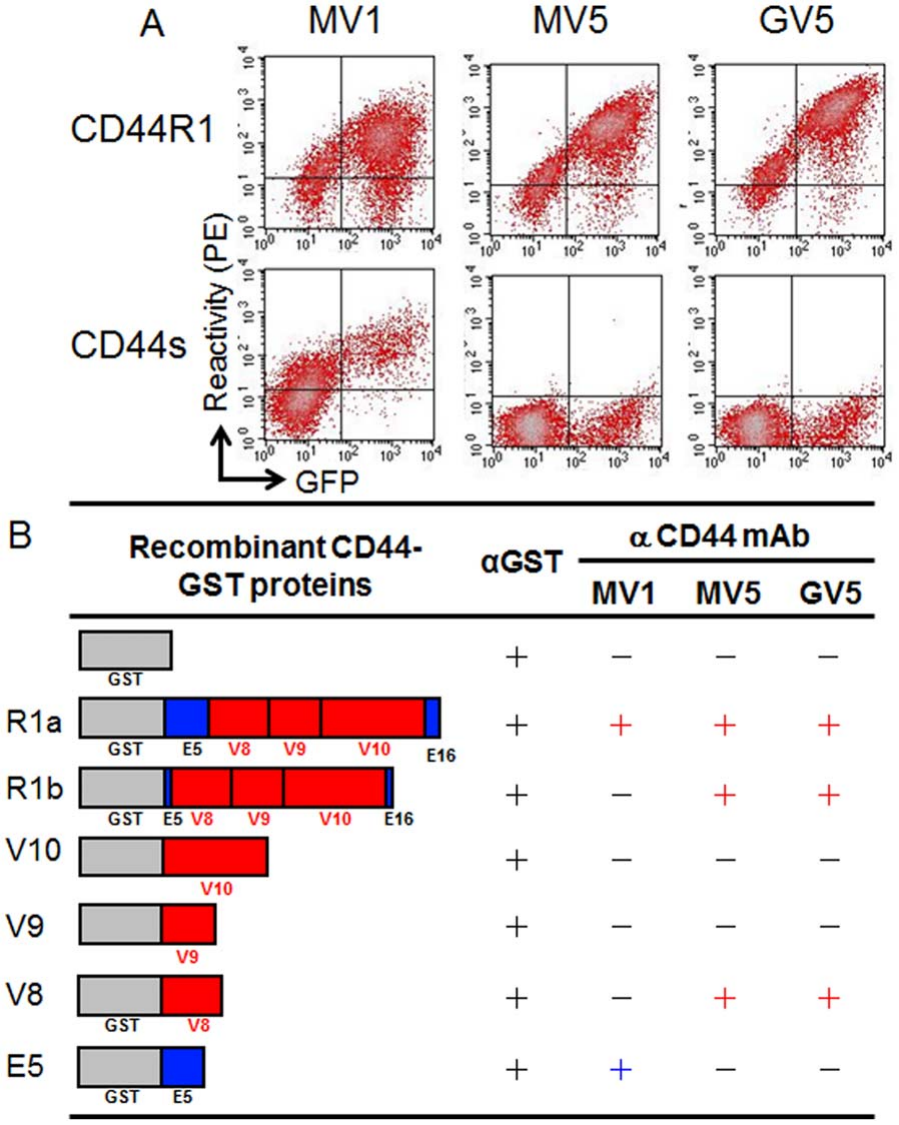
Specificity and epitope of anti-CD44 fully human mAb. (A) MV1 (left), MV5 (middle) and GV5 (right) were compared for the reactivity with HEK293F cells expressing human CD44R1-GFP (upper) or human CD44s-GFP (lower) by FCM. (B) Reactivity of antibodies against various GST-fused recombinant CD44 proteins (R1a and R1b; Δex5-v8-v9-v10-Δex16, v8, v9, v10 and ex5) fused to GST was determined by ELISA. Difference in the length of Δex5 and Δex16 between R1a and R1b recombinant proteins is described in “Materials and Methods”.

Specificities of MV1, MV5 and GV5 were compared for the reactivity with HEK293F human embryonic kidney cells expressing CD44R1 or CD44s (**Fig. 3A**), as described elsewhere [10], [23]. MV5 and GV5 reacted specifically with CD44R1-green fluorescent protein (GFP)-expressing cells in a GFP-expression-dependent manner; however, these mAb did not react with cells expressing CD44s-GFP, demonstrating that GV5 maintains the specificity of MV5 and specifically recognizes a human CD44R1 protein by flow cytometry (FCM). In contrast, MV1 reacted with HEK293F cells expressing CD44s-GFP or CD44R1-GFP. The specificity of human mAb was also substantiated by FCM analysis using RH7777 rat cells transfected with cDNA of human CD44s or CD44R1 (data not shown). Next, MV1, MV5 and GV5 were assessed for reactivity with various recombinant human CD44 proteins fused to glutathione S-transferase (GST) (**Fig. 3B**). R1a is an immunogen for the production of anti-CD44 human mAb, and R1b contains shorter polypeptides in ex5 and v8 regions than R1a. The epitope defined with fully human mAb was localized to an ex5-coding region for MV1, and a v8-coding region for MV5 and GV5 by enzyme-linked immunosorbent assay (ELISA). The peptide epitope defined with MV5 or GV5 seemed to be exposed in human cells because these mAb definitely and specifically reacted with HEK293F cells expressing CD44R1, although CD44R1 is heavily glycosylated and the expression of these epitopes could be affected by the glycosylation varying between cell types or cell conditions.

### Anti-CD44R1 fully human GV5 selectively reacted with human carcinoma cell lines, but not with various human normal cells

**Figure 4:**
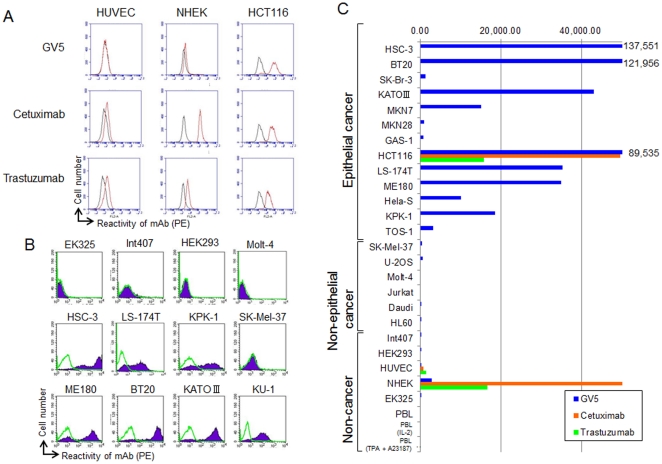
Fine specificity of a class-switched anti-CD44R1 fully human IgG mAb (GV5) revealed by FCM. GV5, Cetuximab and Trastuzumab were compared for the reactivity with HUVEC, NHEK and HCT116 by FCM (histograms) with an Accuri C6 flow cytometer (A). Reactivity of GV5 with various human cells was analyzed by FCM, and depicted as histograms (B) using a BD-LSR flow cytometer and as ΔMFI (C) using an Accuri C6 flow cytometer.

Some of the existing therapeutic mAb are targeted to the human epidermal growth receptor (HER) family [24]–[27]. We compared the reactivity of GV5, Cetuximab (anti-HER1) and Trastuzumab (anti-HER2) with HCT116 human colon cancer cells, normal human epidermal keratinocytes (NHEK) and human umbilical vein endothelial cells (HUVEC) (**Fig. 4A, C**). GV5, Cetuximab and Trastuzumab were definitely reactive with HCT116 human cancer cells. GV5 weakly reacted with NHEK, although Cetuximab strongly and Trastuzumab moderately reacted with NHEK. Furthermore, GV5 was almost unreactive with HUVEC, although Cetuximab and Trastuzumab substantially reacted with HUVEC. Next, we examined the reactivity of GV5 against various additional human cell lines (**Fig. 4B**). GV5 definitely reacted with various cultured human epithelial cancers (BT20 breast, KATOIII stomach, LS-174T colon, ME180 cervix, KPK-1 kidney and KU-1 bladder), although this mAb did not react or only weakly reacted with Molt-4 T leukemia, SK-MEL-37 melanoma and non-cancerous cell lines from adult skin (EK325), fetal intestine (Int407) and embryonic kidney (HEK293F). GV5 also reacted with MKN-7 stomach and HeLa-S uterine cervix carcinoma cell lines, but not with U-2OS osteosarcoma, Jurkat, Daudi and HL60 leukemia cell lines (**Fig. 4C**). Expression of CD44R1 in many carcinoma cells was heterogeneous (**Fig. 4B**), as in the cases of human xenografts in athymic mice (**Fig. 1** **,** **Fig. 2**). In addition, GV5 did not react with resting small lymphocytes at all; it is also of interest that it did not react with lymphocytes stimulated with recombinant human interleukin-2 (IL-2) for 48 h or 12-*O*-tetradecanoylphorbol-13-acetate (TPA) plus Ca ionophore (A23187) for 24 h, indicating that activated T and B lymphocytes are not reactive with GV5 (**Fig. 4C**). The specificity of GV5 indicates that CD44R1 is a tumor-selective and superior target molecule compared with HER1 or HER2. In fact, serious skin toxicity was observed in the treatment of Cetuximab against colon cancer patients, probably because of binding to HER1 on skin keratinocytes. In the context of the expression of HER2, GV5 strongly reacted with a BT20 triple-negative breast cancer cell line that does not express estrogen and progesterone receptors and HER2, although this mAb did not react with HER2-over-expressing SK-BR-3 breast cancer cell line (**Fig. 4B, C**). Therefore, we expect that anti-CD44R1 therapeutic mAb could compensate for anti-HER2 mAb in the therapy of breast cancers.

### CD44R1 was specifically detected in human epithelial cancer tissues

**Figure 5:**
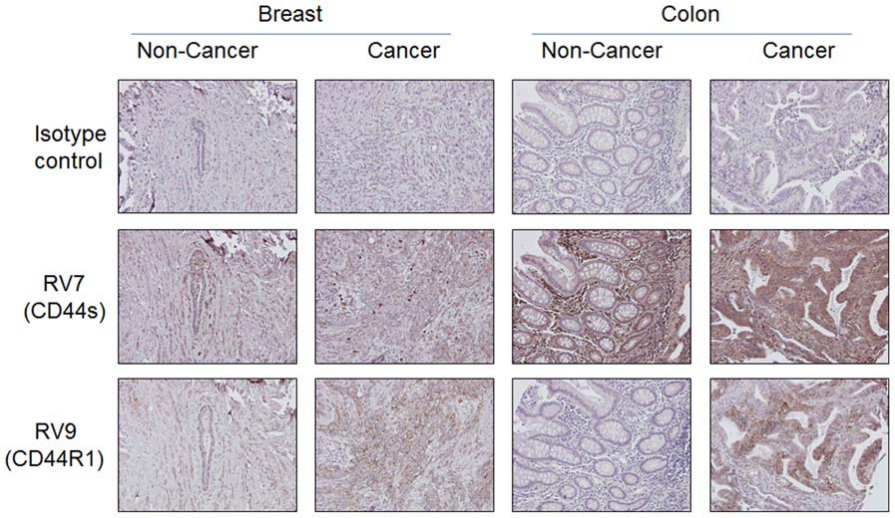
Immunoperoxidase staining of human breast and colon tissues with anti-human CD44 rat mAb. Reactivity of anti-human CD44s or anti-human CD44R1 (v8) rat mAb with breast and colon tissue sections from human surgical specimens was analyzed with ABC immunoperoxidase staining. Sections from PFA-fixed and paraffin-embedded human breast and colon tissues were treated with microwaves in citrate buffer for the retrieval of antigens, and incubated with RV7 or RV9 rat mAb. After being rinsed in PBS, tissue sections were sequentially incubated with species-specific biotinylated donkey anti-rat IgG (H + L), ABC reagent and substrate solution containing DAB and H_2_O_2_. Nuclei were stained with hematoxylin. Non-Cancer or Cancer tissues mean specimens from an identical patient.

**Figure 6:**
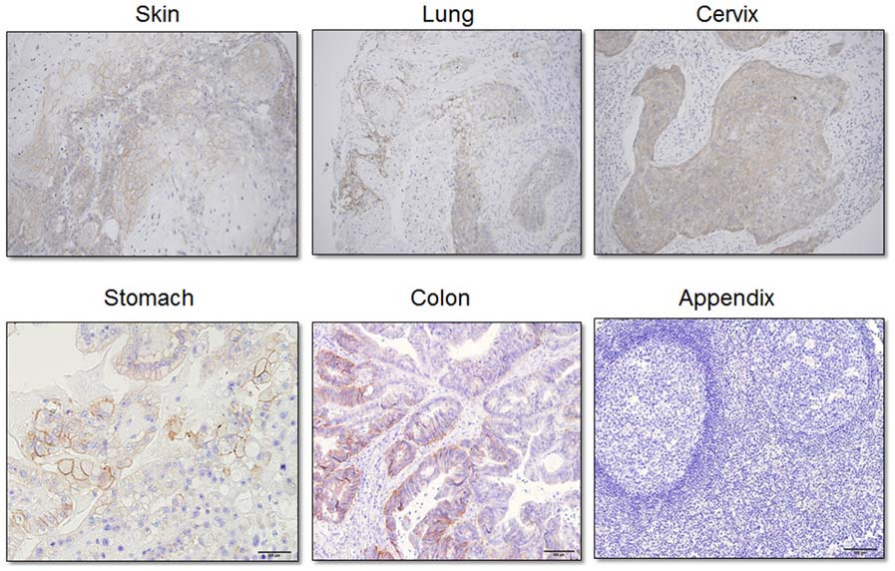
Immunoperoxidase staining of human tissues with anti-human CD44 fully human IgG mAb (GV5). Sections from PFA-fixed and paraffin-embedded human tissues were treated with microwaves in citrate buffer for the retrieval of antigens, and incubated with biotinylated GV5. After being rinsed in PBS, tissue sections were incubated with ABC reagent and substrate solution containing DAB and H_2_O_2_. Nuclei were stained with hematoxylin.

First, the distribution of CD44s and CD44R1 in human cancers was analyzed with rat mAb (RV7 and RV9) against human CD44, since immunostaining of human tissues with GV5 followed by anti-human immunoglobulins did not result in the obvious result (data not shown). In IHA on human breast and colon tissues (**Fig. 5**), both RV7-defined CD44s and RV9-defined CD44R1 (v8) were definitely expressed in the cell membrane of breast and colon cancer cells, although CD44s but not CD44R1 was also expressed on cells in the stroma. Expression of CD44R1 in normal breast epithelial cells was low and was negative in colon epithelial cells. In addition, CD44R1 was heterogeneously expressed in cancer cells, as in the case with the staining of human cancer xenografts (**Fig. 1** **and** **Fig. 2**) and cancer cell lines (**Fig. 4B**) by GV5 anti-CD44R1 human mAb. In IHA on human tonsils, RV9 anti-CD44R1 (v8) mAb weakly stained the epithelium, especially cells in the basal layer, but did not stain lymphoblastoid cells in the germinal center of the tonsil tissue, although RV7 anti-CD44s mAb definitely stained cells in this region, in addition to cells in the whole epithelium (data not shown). These findings coincide well with the unreactivity of GV5 with activated human lymphocytes (**Fig. 4C**). These results from FCM and IHA have demonstrated excellent cancer-specificity of CD44R1, undoubtedly superior to that of CD44s. We next examined the reactivity of biotinylated GV5 with human tissue specimens. GV5 definitely reacted with squamous carcinomas of uterine cervix (primary cancer), skin (primary cancer) and lung (metastatic cancer to soft tissues), adenocarcinomas of stomach and colon, but not with aggregated lymphatic nodules of vermiform appendix (**Fig. 6**). Thus, the expression of CD44R1 is limited to epithelial cancers, and is not elevated in the process of lymphocyte activation *in vitro* or *in vivo*, although the expression of a given intrinsic oncoprotein in lymphocytes is often up-regulated by various activation stimuli [28], [29].

### Anti-CD44R1 GV5 human mAb exhibited *in vivo* therapeutic effect on human carcinomas in xenograft models

**Figure 7:**
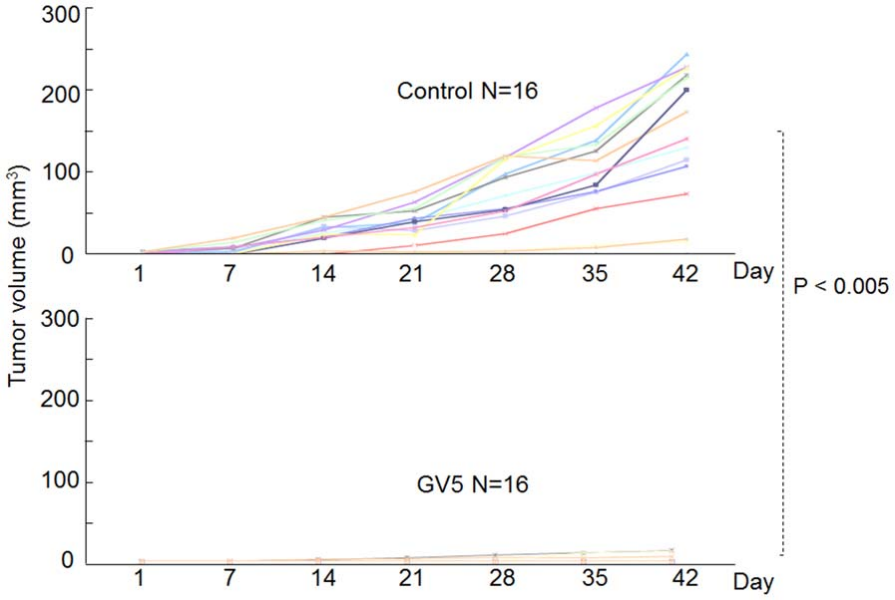
Therapeutic effect of anti-CD44R1 fully human mAb in the tumor neutralization model. Tumor neutralization model was adopted. ME180 human tumor cells (1.0×10^6^) with or without mAb (50 µg/site) in 200 µl of PBS were subcutaneously inoculated into the right dorsal flank of each animal. The size of each tumor formed was periodically measured, and tumor volume (mm^3^) was calculated by the formula 0.4×(length)×(width)^2^. Results were analyzed statistically by two-way ANOVA tests with repeated measures.

**Figure 8:**
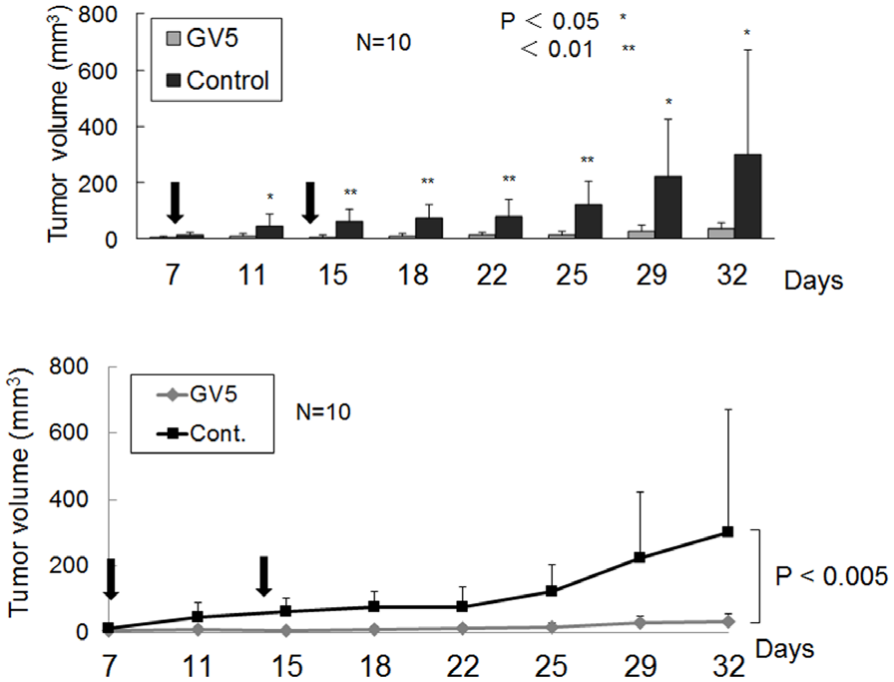
Therapeutic effect of anti-CD44R1 fully human mAb in the established tumor model. Established tumor model was adopted. Seven days after human larynx carcinoma-derived HSC-3 tumor cells (1.0×10^6^) in 200 µl of PBS were inoculated subcutaneously to athymic mice and visible tumor in each mouse was confirmed, 500 µl of PBS with or without GV5 (100 µg) was intraperitoneally inoculated at day 7 and day 14 (vertical arrows). The size of each tumor formed was periodically measured, and tumor volume (mm^3^) was calculated by the formula 0.4×(length)×(width)^2^. Results were analyzed statistically by two-sided Student's *t* tests (upper), and by two-way ANOVA tests with repeated measures (lower). Vertical bars show standard deviations.

GV5 was evaluated for anti-tumor effect against human tumors in athymic mice. The size of each tumor formed was periodically measured, as described elsewhere [30]–[32]. First, GV5 was examined for the effect on tumor formation by ME180 human cervix cancer cells in a tumor neutralization model [33]. This model to evaluate the local effect of antibodies against tumor growth historically originated from Winn's test [34] intended for evaluation of the anti-tumor effect of cytotoxic T lymphocytes. Cancer cells were inoculated subcutaneously to athymic mice with or without GV5 (50 µg/site), and tumor growth in GV5 treatedmice was significantly inhibited compared with that of control mice (**Fig. 7**). Next, GV5 was examined for anti-tumor effect against an HSC-3 human larynx cancer in an established tumor model [35]. In this systemic administration of mAb to tumor-bearing mice, we deliberately adopted intraperitoneal but not intravenous injection for the administration of an exact amount of mAb to each mouse. Seven days after HSC-3 cells were inoculated subcutaneously to athymic mice and a visible tumor in each mouse was confirmed, GV5or vehicle control was intraperitoneally injected twice at an interval of a week. In this experimental model, tumor growth in GV5 treatedmice was again significantly inhibited compared with that of control mice (**Fig. 8**).

### GV5 induced internalization of CD44R1 and ADCC *in vitro*

**Figure 9:**
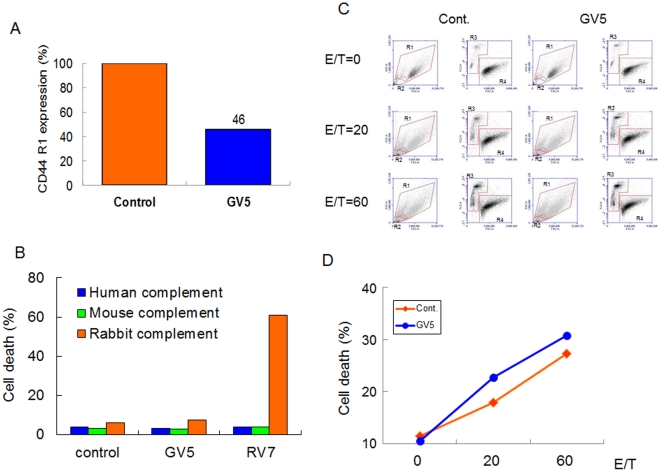
Internalization of CD44R1, CDC and ADCC by GV5 fully human mAb. (A) HSC3 cells were cultured with or without GV5 (10 µg/ml) for 12 h, and were immunostained with GV5 followed by FITC-conjugated donkey anti-human IgG (H+L). Cell-surface CD44R1 proteins in these cells were detected by FCM. Experiments were repeated three times, and similar results were obtained. (B) After HSC-3 cells and sera for complement and mAb were mixed and incubated in each well of U-bottomed 96-well plate for 1 h, DAPI was added to each well. Percentages of DAPI-stained cells (dead cells) were calculated by FCM. Experiments were repeated three times, and similar results were obtained. (C) HSC-3 cells (1.5×10^5^) were mixed with effector cells of splenocytes (3×10^6^ or 9×10^6^) from KNS nude mice, which were pre-cultured overnight with lL-2, with or without mAb, in each well of U-bottomed 96-well plate for 5 h, and PI was added to each well. Cytotoxicity was analyzed using an Accuri flow cytometer. **R1**, HSC3 human target cells; **R2**, mouse effector cells; **R3**, PI-stained dead cells in **R1**; **R4**, PI-unstained living cells in **R1**. (D) Cell death (%) of HSC-3 cells by mouse effector cells (E/T = 0, 20 or 60) with or without GV5 was plotted. E/T means effector to target ratio.

To analyze the mechanisms of the *in vivo* anti-tumor effect of GV5 against human cancer xenografts in athymic mice, internalizations of CD44R1, complement-dependent cytotoxicity (CDC) and antibody-dependent cellular cytotoxicity (ADCC) by GV5 were examined. Since the dysfunction of CD44R1 by mAb might lead to growth inhibition or cell death of cancer cells through a lack of signal from an adhesion substrate, we examined the effect of GV5 on the distribution of cell-surface CD44 proteins. By the addition of GV5 to the culture of HSC-3 cells for 12 h, approximately 50% of CD44R1 proteins disappeared from the cell surface (**Fig. 9A**). CDC using GV5 combined with mouse, rabbit or human serum did not result in the death of tumor cells (**Fig. 9B**). In ADCC, splenocytes of athymic mice were pre-cultured with IL-2 for 12 h to enhance the killing activity of effector cells. By the use of IL-2-treated effector cells, GV5clearly augmented the cytotoxicity against HSC-3 tumor cells in ADCC (**Fig. 9C, D**).

### GV5 induced efficient ADCC with human effector cells

**Figure 10:**
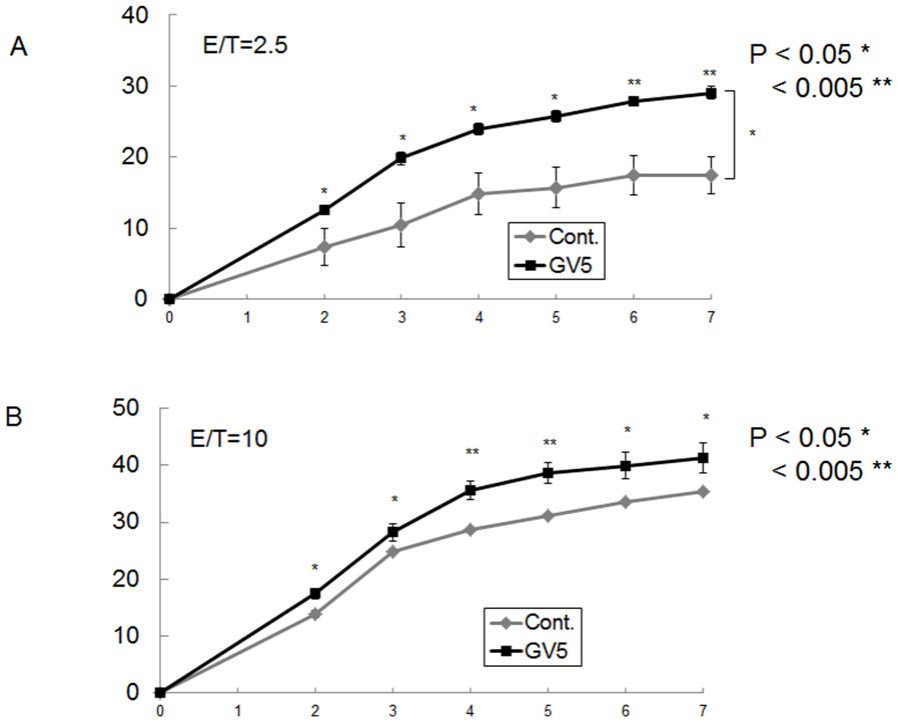
ADCC activity of anti-CD44R1 fully human mAb against human tumor cells using human lymphocytes. HSC-3 cells were labeled with Calcein-AM, and cells (2×10^5^) were mixed with effector cells of human PBL (5×10^5^ or 2×10^6^), which were pre-cultured overnight with IL-2 with or without mAb in each well of U-bottomed 96-well plate for 7 h. Cytotoxicity was evaluated by the release of Calcein-AM by dead tumor cells into the medium, and results were automatically recorded using a Terascan VP microfluorocytometer at 1 h intervals for 7 h. Results were analyzed statistically by two-way ANOVA tests with repeated measures (A) and by two-sided Student's *t* tests (A and B). Spots and vertical bars respectively show means and standard errors.

Since we confirmed ADCC activity of GV5 with mouse effector cells, we next examined ADCC activity of GV5 with human effector cells. GV5 showed significant ADCC activity against HSC-3 tumor cells with IL-2-stimulated human peripheral blood lymphocytes (PBL) as effector cells, even at a low effector/target (E/T) ratio of 2.5 (**Fig. 10A**). At an E/T ratio of 10, GV5 showed augmented cytotoxicity against HCS-3 cells compared with human effector cells alone, at all time points between 2 h and 7 h (**Fig. 10B**).

In this study, we have successfully produced fully human GV5 mAb specifically recognizing CD44R1, and demonstrated the growth inhibition of human cancer xenografts in athymic mice by GV5. As to the anti-tumor mechanisms against human xenografts by GV5 in athymic mice, we tentatively propose the contribution of induced internalization of CD44R1 by GV5 and augmented cytotoxicity in ADCC by mouse effector cells with GV5. We also expect that GV5can display an ADCC-mediated therapeutic effect on human malignancies, since GV5has showed ADCC activity with human PBL as effector cells. Effects of anti-CD44R1 mAb on the incorporation of hyaluronate are now under investigation; however, we have already confirmed that GV5 could induce internalization of CD44R1 from the cell surface, suggesting possible effects on the incorporation of hyaluronate and augmented anti-tumor effect of this mAb, in addition to ADCC activity.

Recently, specific chimeric or humanized mAb against the extracellular domains of HER2 [24], [25], HER1 [26], [27] and CD20 [36]–[38] have been introduced for the treatment of breast cancer, colorectal cancer or B cell malignancies, respectively. Although phase I clinical trials with anti-CD44v6 chimeric mAb against squamous cell carcinomas have recently been performed [39]–[41], serious skin toxicity was observed probably because of binding to CD44v6 on skin keratinocytes [39], [40]. In this context, reactivity of GV5 with human normal skin keratinocytes was negative or negligible (**Fig. 3A**), suggesting low skin toxicity of our anti-CD44R1 (v8) fully human mAb.

Accumulated evidence has shown that CD44 is characteristically expressed in CSCs of various tissue origins [11]–[16]. We now focus our attention on CD44v (CD44R1) but not CD44s expressed on the surface of CSCs in the precancerous region of gastric adenocarcinomas of *K19-Wnt1/C2mE* transgenic mice [9], [10]. Reactivity of GV5 with ME180 or HSC-3 seemed relatively heterogeneous; however, tumor formation or tumor growth was almost completely inhibited by GV5, suggesting that CSCs are mainly composed of GV5-reactive CD44R1^high^ cells but not of GV5-unreactive CD44R1^low^ cells. In this context, our recent data have provided evidence that the expression of CD44R1 and its association with xCT cysteine-glutamate antiporter, a light chain subunit of CD98 oncoprotein [10], [23], [30]–[32], [42], [43], block the reactive oxygen species (ROS)-induced stress signaling that results in growth arrest, cell differentiation and senescence, and thereby promote the proliferation of cancer cells and the formation of lethal gastrointestinal tumors [44]. Given that CD44R1-expressing CSCs play a central role in resistance to cancer therapy, it should be postulated that therapeutic mAb could target the CD44R1^high^ cell population in cancer.

Our present analyses strongly indicate that cancer therapy with anti-CD44R1 fully human mAb is promising, especially against various human epithelial cancers such as adenocarcinomas of breast, stomach and colon, transitional cell carcinoma of bladder, and renal carcinoma, in addition to squamous cell carcinomas from various body regions.

## Materials and Methods

### Cells and cell-culture conditions

Cell lines from human larynx carcinoma (ca) (HSC-3), breast ca (BT20, SK-Br-3), gastric ca (GAS-1, MKN-7, MKN-28, KATOIII), colorectal ca (LS-174T, HCT116), uterine cervix ca (ME180, HeLa-S), renal ca (KPK-1, TOS-1), bladder ca (KU-1), melanoma (SK-Mel-37), osteosarcoma (U-2OS), fetal intestine (Int407) and a rat hepatoma cell line (RH7777; generously provided by Tanabe Mitsubishi Pharm.) were cultured in Dulbecco's modified Eagle's medium (DMEM; Sigma-Aldrich, St. Louis, MO, USA) supplemented with 7% heat-inactivated fetal bovine serum (FBS; ICN Biomedicals, Aurora, OH, USA) in a humidified incubator (5% CO_2_) at 37°C. GAS-1, MKN-28 or TOS-1 was respectively obtained from Kotanagi H (Akita University), Japanese Collection of Research Bioresources (JCRB) Cell Bank or Satoh M (Tohoku University). T-cell leukemias (Jurkat, Molt-4), B-cell leukemia (Daudi), promyelocytic leukemia (HL60) of human origin, human PBL with or without recombinant human interleukin 2 (IL-2, 100 IU; Shionogi & Co. Ltd., Osaka, Japan) and mouse splenocytes with IL-2 (100 IU) for ADCC, mouse myelomas (SP2, X63) and established hybridoma cells were cultured in RPMI 1640 medium (RPMI; Sigma-Aldrich) supplemented with 7% heat-inactivated FBS under the conditions mentioned above. To obtain activated human lymphocytes for FCM, PBL were also cultured and stimulated with IL-2 (100 IU) for 48 h or 12-*O*-tetradecanoylphorbol-13-acetate (TPA, 20 ng/ml; Sigma-Aldrich) plus Ca ionophore (A23187, 0.3 µM; Calbiochem, La Jolla, CA, USA) for 24 h in RPMI with 7% FBS. HUVEC (Takara Bio Inc., Otsu, Japan) were maintained in EGM-2 medium (Lonza, Walkersville, MO, USA) and used at third to fifth passages. NHEK (Takara Bio Inc.) were cultured in HuMedia-KG2 (Lonza) and used at third to fourth passages. HEK293F human embryonic kidney cells (Invitrogen, Carlsbad, CA, USA) and a human epidermal keratinocyte-derived cell line (EK325) established by us were cultured in FreeStyle293 expression medium (Invitrogen) in a humidified incubator (5% CO_2_). GFP was genetically fused to the cytoplasmic carboxyl terminus of human CD44s or CD44R1 in a pAcGFP expression vector (BD Biosciences, Mountain View, CA, USA), and HEK293F and RH7777 cells were respectively transfected with these CD44-GFP plasmids by 293fectin (Invitrogen) or Lipofectamine 2000 (Invitrogen), in accordance with the manufacturer's instructions, selected using culture media containing G418 (Nacalai Tesque, Kyoto, Japan) diluted to 400 µg/ml, and clone-sorted for cellular green fluorescence using a JSAN cell sorter (Bay Bioscience, Kobe, Japan). These established HEK293F and RH7777 cell lines expressing CD44-GFP proteins were maintained in FreeStyle293 expression medium with G418 (400 µg/ml). Cells were obtained from American Type Culture Collection (ATCC), unless otherwise stated. Most cell lines containing HSC-3 were mentioned elsewhere [45].

### Existing therapeutic monoclonal antibodies

Anti-HER1 chimeric Cetuximab (MerckSerono, Rockland, MA, USA), anti-HER2 humanized Trastuzumab and anti-CD20 chimeric Rituximab (Roche-Chugai, Tokyo, Japan) were used for the staining of human cells or tissues.

### Preparation of a recombinant fully human IgG1 mAb recognizing CD44R1

MV1, MV2, MV3, MV4 and MV5 fully human IgM mAb were prepared from cell fusion between spleen cells of Kirin-Medarex (KM) mice (22) immunized against a recombinant Δex5-v8-v9-v10-Δex16 (R1a) protein (8) fused to glutathione S-transferase (GST) and SP2 mouse myeloma cells. The procedure for the cell fusion and establishment of hybridomas was performed as described in the next section. In R1a recombinant proteins, Δex5 or Δex16 respectively corresponds to a partial short peptide (Δex5, 19 amino acids; Δex16, 8 amino acids) adjacent to v8 or v10. Reverse-transcribed cDNAs from the variable region of heavy and light chains, which were cloned from total RNA of hybridoma cells secreting an IgM (μ, κ human mAb against CD44R1 (MV5), were genetically reshaped to cDNAs of human IgG1 (γ1, κ), and subcloned to a pBud4.1 expression vector (Invitrogen) having two separate cloning sites with independent promoters. This expression vector was transfected into HEK293F cells with a 293fectin reagent. Two days later, secreted antibodies in the culture medium were assessed for binding to CD44R1 proteins by FCM. Culture medium (two liters) containing human IgG against CD44R1 was concentrated to about 20 ml using a concentration apparatus (Millipore, Billerica, MA, USA) and precipitated by 50%-saturated ammonium sulfate. Further purification was performed by affinity chromatography with protein G-conjugated Sepharose (GE Healthcare, Uppsala, Sweden). Purity of GV5 human IgG mAb was determined by SDS-PAGE analysis followed by protein staining with Coomassie Brilliant Blue (CBB; ICN Biomedicals). Biotinylation of GV5 was performed using EasyLink Biotin conjugation kit (Abcam, Tokyo, Japan), in accordance with the manufacturer's instructions.

### Rat mAb recognizing CD44R1 and CD44s

Female F344/N rats were administered subcutaneous and intraperitoneal injections (first and second immunizations) followed by a final intravenous injection of RH7777 cells expressing human CD44R1-GFP (1.0 to 5.0×10^7^) in each immunization at 3-week intervals. The immune spleen cells (1.0×10^8^) were fused with X63 mouse myeloma cells (2.5×10^7^) using 50% polyethylene glycol 1540 (Roche, Penzberg, Germany). After the cell fusion, hybridoma cells were selected in RPMI supplemented with hypoxanthine, aminopterin and thymidine (50× HAT; Invitrogen). From 960 hybridoma cultures, RV7 (γ2b, κ) anti-human CD44s, RV9 (γ2b, κ) anti-human CD44v8 and RV3 (γ2a, κ) anti-human CD44v9 rat mAb were selected, and RV7 and RV9 were used in this study. Purification of mAb was performed with Protein G-conjugated Sepharose from ascitic fluids precipitated using 50%-saturated ammonium sulfate. Purity of rat IgG mAb was determined by SDS-PAGE analysis followed by protein staining with CBB.

### Determination of the epitope region recognized by anti-CD44 mAb

Reactivity of mAb against various GST-fused recombinant CD44 proteins, R1a and R1b (Δex5-v8-v9-v10-Δex16), v8, v9, v10 and ex5 produced in *Escherichia coli*, was determined by enzyme-linked immunosorbent assay (ELISA). In Δex5-v8-v9-v10-Δex16 (R1b) recombinant proteins, Δex5 or Δex16 respectively corresponds to a partial short peptide (Δex5, 3 amino acids; Δex16, 2 amino acids) adjacent to v8 or v10. Aliquots (50 µl) of antigens diluted to 20 µg/ml in phosphate-buffered saline (PBS) were passively adsorbed in triplicate in wells of polyvinylchloride 96-well plates (Sumitomo Bakelite, Tokyo, Japan) overnight at 4°C. Each well was treated with 100 µl of Block Ace (Dainihon Seiyaku, Osaka, Japan) for 3 h at 37°C to inhibit nonspecific binding of the staining reagents. Anti-CD44 human IgM mAb (hybridoma culture supernatant) was used without dilution, and GV5 human IgG, RV3, RV7 and RV9 rat IgG were diluted to 10 µg/ml in PBS containing 1% bovine serum albumin (BSA; ICN Biomedicals). Fifty µl of anti-CD44 mAb or anti-GST goat polyclonal antibody (GE Healthcare; 1∶300 diluted in 1% BSA-PBS) was added and incubated for 1 h at 37°C. Following intensive washing with PBS containing 0.05% Tween 20 (T-PBS), 50 µl of 1∶1000 diluted species-specific horseradish peroxidase (HRP)-conjugated donkey anti-human, anti-rat or anti-goat IgG (H+L) (Jackson ImmunoResearch, West Grove, PA, USA) was added to each well and incubated for 60 min at room temperature. After wells were washed intensively with T-PBS, they were supplemented with 0.1 M citric-acetate buffer (pH 6.0) containing 3, 3′, 5, 5′-tetramethylbenzidine (0.1 mg/ml) and 0.01% H_2_O_2_ (50 µl in each well). Color development in the wells was stopped after 5 to 10 minutes by the addition of 0.5 M H_2_SO_4_ (75 µl in each well), and the optical density of the solution at 450 nm was measured with a Model 550 microplate reader (Bio-Rad, Hercules, CA).

### Flow cytometry (FCM)

For cell-surface staining, cells (3.0×10^5^) in 50 µl of PBS containing 1% BSA were mixed with primary antibodies (50 µl) of undiluted hybridoma culture supernatant or purified mAb diluted to 10 µg/ml in 1% BSA-PBS and incubated for 1 h at 4°C. After three washes with PBS, cells were incubated at 4°C for 30 min in 50 µl of 1∶200 diluted goat anti-human IgG and IgM (H+L) antibodies labeled with fluorescein isothiocyanate (FITC) or phycoerythrin (PE) (Jackson ImmunoResearch). After three washes with PBS, cells were suspended in PBS containing 0.2% BSA, and analyzed with an Accuri C6 flow cytometer (Tomy Digital Biology, Tokyo, Japan) or a BD-LSR flow cytometer (Becton-Dickinson, Sunnyvale, CA, USA). For the quantification of the expression of cell-surface antigen, subtracted mean fluorescence intensity (ΔMFI) was calculated.

### Immunohistological analysis (IHA)

Tissue sections (5 µm thick) of ME180 human cancer xenografts, which developed in athymic mice and were snap-frozen in OCT embedding medium (Sakura Fineteck, Tokyo, Japan) using liquid nitrogen, were prepared using a CM1800 cryostat (Leica Microsystems, Wetzlar, Germany). Sections were fixed in 4% paraformaldehyde (PFA) in PBS or cold acetone, treated with Block Ace (Dainihon Seiyaku) for 1 h, and incubated with hybridoma culture supernatant (MV1, MV2, MV3, MV4 or MV5 human mAb) diluted 1∶3 with 1% BSA-PBS or humanized mAb (GV5, Trastuzumab or Rituximab) diluted to 10 µg/ml with 1% BSA-PBS overnight at room temperature. Human tissue specimens on various cancer tissue arrays (BB6; Super Bio Chip Laboratories, Seoul, Korea) and gastric and colon cancers and appendix (these specimens were collected with informed consent from gastric or colon cancer patients who underwent surgery at Kumamoto University Hospital and informed consent was obtained with documents) were treated with microwaves (500 W) for 10 min in citrate buffer (pH6.0) for the retrieval of antigens, treated with Block Ace for 1 h and incubated with RV7, RV9 or biotinylated GV5 mAb (10 µg/ml diluted in 1% BSA-PBS) overnight at room temperature. After washing with PBS, endogenous peroxidase activity of tissues was inhibited by immersing sections in 3% H_2_O_2_-methanol for 5 min. After being rinsed in PBS, tissue sections were incubated with biotinylated goat anti-human IgG and IgM (H+L), biotinylated goat anti-human IgG Fcγ or biotinylated donkey anti-rat IgG (H+L) (Jackson ImmunoResearch) diluted 1∶1000 in 1% BSA-PBS for 1 h. This step was skipped in the case of the staining with biotinylated GV5. After three washes with PBS, samples were treated with avidin-biotin-peroxidase complex (ABC) reagent (Vector Laboratories, Burlingame, CA, USA) diluted 1∶200 in 0.1% BSA-PBS for 45 min. After three more washes with PBS, tissue sections were incubated with 0.05% 3,3′-diaminobenzidine (DAB; Dojin Chemicals, Kumamoto, Japan) and 0.01% H_2_O_2_ in 0.1 M Tris-HCl (pH 7.4), and counterstained with hematoxylin. Samples were dehydrated with ethanol (50%, 70%, 75%, 90%, 95% and 99%, successively), cleared in xylene and mounted in Permount (Fisher Scientific, Fair Lawn, NJ, USA). The location of antibody-defined components was observed under an Axiolab microscope (Zeiss, Hamburg, Germany) and photographed.

### Evaluation of therapeutic effect of an anti-CD44R1 mAb on human tumors in athymic mice

Male athymic mice (KSN strain) at 6 weeks of age (Shimizu Animal Farm, Kyoto, Japan), housed in a controlled environment at 22°C in a specific-pathogen-free facility, were randomly distributed into two groups (with or without treatment of mAb). Adherent human tumor cells in culture dishes were trypsinized and washed with PBS three times and re-suspended in PBS. In the first experiment, ME180 human tumor cells (1.0×10^6^) with or without mAb (50 µg/site) in 200 µl of PBS were subcutaneously inoculated into the right dorsal flank of each animal (tumor neutralization model [33]). In the second experiment, aliquots of 200 µl of the cell suspensions of HSC-3 human tumor cells (1.0×10^6^) were subcutaneously inoculated into the right dorsal flank of each animal (day 0), and 500 µl of PBS with or without mAb (100 µg) was intraperitoneally injected at day 7 and day 14 (established tumor model [35]). The size of each tumor formed was periodically measured, and tumor volume (mm^3^) was calculated by the formula 0.4×(length)×(width)^2^ [30]–[32]. All mice were used with the approval (approval IDs: KAPS-19-039, -042, -043 and KAPS-22-004) of the Committee for the Care and Use of Laboratory Animals at Kinki University.

### Internalization of CD44 proteins by the treatment with mAb

HSC-3 cells were cultured for 12 h with or without GV5 (10 µg/ml) at 37°C, and incubated with FITC-conjugated donkey anti-human IgG (H+L) antibodies (Jackson ImmunoResearch) for 1 h on ice. Expression of cell-surface CD44R1 proteins in these cells was analyzed by FCM with a BD-LSR flow cytometer.

### Complement-dependent cytotoxicity (CDC)

Target cells (1.5×10^5^), sera as complement source (1∶10 diluted) and mAb (10 µg/ml) at the final concentration were incubated in each well of U-bottomed 96-well plate (Nunc-Thermo Scientific) for 1 h at 37°C, and 4,6′-diamidino-2-phenylindole (DAPI; Sigma-Aldrich) was added to each well (0.5 µg/ml). Percentages of DAPI-stained cells (dead cells) were calculated by FCM with a BD-LSR flow cytometer.

### Antibody-dependent cellular cytotoxicity (ADCC)

For mouse effector cells, ADCC was assessed as follows. HSC-3 cells (2×10^5^) were mixed with splenocytes (4×10^6^ or 1.2×10^7^) of KNS nude mice, which were pre-cultured for 12 h with recombinant human interleukin 2 (IL-2, 100 IU; Shionogi & Co. Ltd., Osaka, Japan) in 650 µl of RPMI with or without mAb (10 µg/ml) in each well of 12-well plate (Sumitomo Bakelite; Suspension Culture Treated) for 5 h at 37°C, and propidium iodide (PI; Sigma-Aldrich) was added to each well (0.5 µg/ml). Percentages of PI-stained cells (dead cells) were calculated by FCM with an Accuri C6 flow cytometer. For human effector cells, ADCC was assessed using a Terascan VP microfluorocytometer (Minerva Tech Co., Tokyo, Japan). HSC-3 (2×10^4^ cells) in DMEM with 7% FBS (200 µl) were cultured on wells of a 96-well flat-bottomed microplate (Costar 3596; Corning Inc., Corning, NY) for 24 h. The monolayer HSC-3 cells were stained using Calcein-AM (Dojin Chemicals) at a concentration of 10 µg/ml at 37°C for 1 h. Following gentle washing of cells attached to the bottom of wells, human PBL, which were pre-cultured with IL-2 (100 IU) for 12 h, were added (5×10^4^ or 2×10^5^ cells/well) with or without GV5 at a final concentration of 1 µg/ml. After the distribution of the materials into wells, the plate was centrifuged at 100×*g* for 2 min to cause the effector cells to settle. DMEM with 7% FBS was added to empty wells (200 µl/well) for measurement of a background fluorescence level. All sets of measurements were carried out in triplicate wells. Fluorescence intensity of each well was measured by focusing an inverted epifluorescent microscope equipped with Terascan VP on a cell surface and adjusting the sensitivity of a photomultiplier. Thereafter, 10 µl of Decon 90 (Decon Laboratories, Hove, UK) was added to a well for the maximal release measurement for entirely injured cells. The plate was incubated for the indicated periods up to 7 h at 37°C. The extent of Calcein-AM release in the experimental wells was automatically calculated and is expressed as the percentage of cytotoxicity.

### Statistical analyses

We analyzed results statistically by two-sided Student's *t* tests with Microsoft Excel 2007 (Microsoft, Redmond, WA), and/or by two-way analysis of variance (ANOVA) tests with repeated measures using R statistical package (www.r-project.org).
